# Chloridobis(dimethyl­glyoximato-κ^2^ *N*,*N*′)(4-methyl­pyridine-κ*N*)cobalt(III) hemihydrate

**DOI:** 10.1107/S1600536811020162

**Published:** 2011-06-04

**Authors:** Madhavan Amutha Selvi, Kannan Arun Kumar, Arunachalam Dayalan

**Affiliations:** a Department of Chemistry, Loyola College (Autonomous), Chennai 600 034, India

## Abstract

In the title complex, [Co(C_4_H_7_N_2_O_2_)_2_Cl(C_6_H_7_N)]·0.5H_2_O, the central Co^III^ ion, chelated by four N atoms of the two bidenate glyoximate ligands, exhibits a slightly distorted octa­hedral geometry. The axial positions are occupied by a chloride ion and the 4-methyl­pyridine N atom. Inter­molecular O—H⋯O hydrogen bonds link the mol­ecules in the crystal *via* the water mol­ecules, while the glyoximate ligands exhibit intra­molecular O—H⋯O hydrogen bonds.

## Related literature

For similar structures, see: Revathi *et al.* (2009 ▶); Kavitha *et al.* (2008 ▶). For vitamin-B_12_ models, see: Brown *et al.* (2005 ▶); Randaccio *et al.* (1989 ▶). For structure–property relationships, see: Gupta *et al.* (2004 ▶); Dutta *et al.* (2009 ▶). For intra­molecular hydrogen bonding, see: Reemers & Englert (2002 ▶); Dolphin (1982 ▶); For details of the synthesis, see: Ramesh *et al.* (2008 ▶); Toscano *et al.* (1983 ▶). For spectroscopic details, see: Dayalan & Vijayaraghavan (2001 ▶); Silverstein & Bassler (1984 ▶); Bline & Hadzi (1958 ▶). For chemical properties of cobaloximes, see: Schrauzer & Windgassen (1966 ▶).
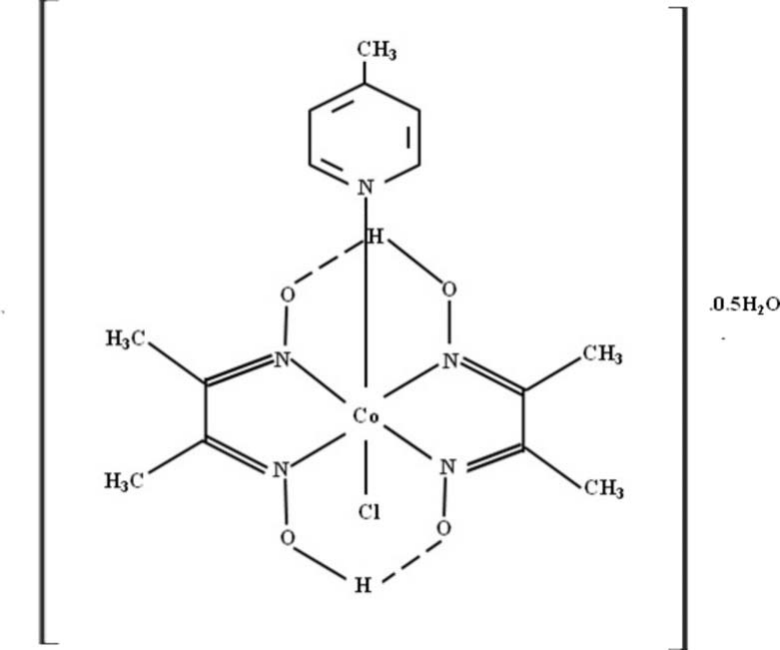


## Experimental

### 

#### Crystal data

[Co(C_4_H_7_N_2_O_2_)_2_Cl(C_6_H_7_N)]·0.5H_2_O
*M* *_r_* = 425.74
Orthorhombic, 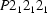

*a* = 8.330 (5) Å
*b* = 14.365 (5) Å
*c* = 15.634 (5) Å
*V* = 1870.8 (14) Å^3^
*Z* = 4
Mo *K*α radiation
μ = 1.09 mm^−1^
*T* = 293 K
0.25 × 0.20 × 0.20 mm

#### Data collection

Bruker SMART APEXII CCD diffractometer
Absorption correction: multi-scan (*SADABS*; Bruker 2008 ▶) *T*_min_ = 0.772, *T*_max_ = 0.811
10519 measured reflections
4642 independent reflections
4143 reflections with *I* > 2σ(*I*)
*R*_int_ = 0.028

#### Refinement

*R*[*F* ^2^ > 2σ(*F* ^2^)] = 0.029
*wR*(*F* ^2^) = 0.077
*S* = 1.02
4642 reflections
248 parameters
2 restraints
H atoms treated by a mixture of independent and constrained refinement
Δρ_max_ = 0.40 e Å^−3^
Δρ_min_ = −0.38 e Å^−3^
Absolute structure: Flack (1983 ▶), 1983 Friedel pairs
Flack parameter: 0.014 (13)

### 

Data collection: *APEX2* (Bruker, 2008 ▶); cell refinement: *APEX2* and *SAINT-Plus* (Bruker, 2008 ▶); data reduction: *SAINT-Plus* and *XPREP* (Bruker, 2008 ▶); program(s) used to solve structure: *SIR92* (Altomare *et al.*, 1993 ▶); program(s) used to refine structure: *SHELXL97* (Sheldrick, 2008) ▶; molecular graphics: *ORTEP-3* (Farrugia, 1997 ▶) and *Mercury* (Macrae *et al.*, 2006 ▶); software used to prepare material for publication: *SHELXL97*.

## Supplementary Material

Crystal structure: contains datablock(s) I, global. DOI: 10.1107/S1600536811020162/jh2292sup1.cif

Structure factors: contains datablock(s) I. DOI: 10.1107/S1600536811020162/jh2292Isup2.hkl

Additional supplementary materials:  crystallographic information; 3D view; checkCIF report

## Figures and Tables

**Table 1:** Selected interatomic distances (Å)

O1⋯O5	2.911 (8)
O5⋯O3^i^	3.013 (7)

Symmetry code: (i) 
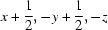
.

**Table 2:** Hydrogen-bond geometry (Å, °)

*D*—H⋯*A*	*D*—H	H⋯*A*	*D*⋯*A*	*D*—H⋯*A*
O3—H3⋯O1	0.90 (1)	1.61 (1)	2.499 (3)	168 (3)
O2—H2⋯O4	0.91 (1)	1.60 (2)	2.483 (3)	162 (4)

**Fig. 1.**
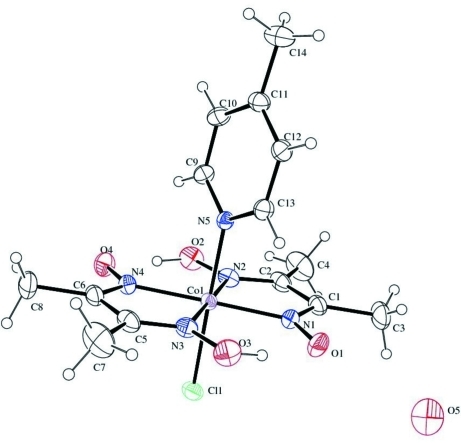
The ORTEP representation of the complex drawn at 30% probability level with the atom labelling scheme.

**Fig. 2.**
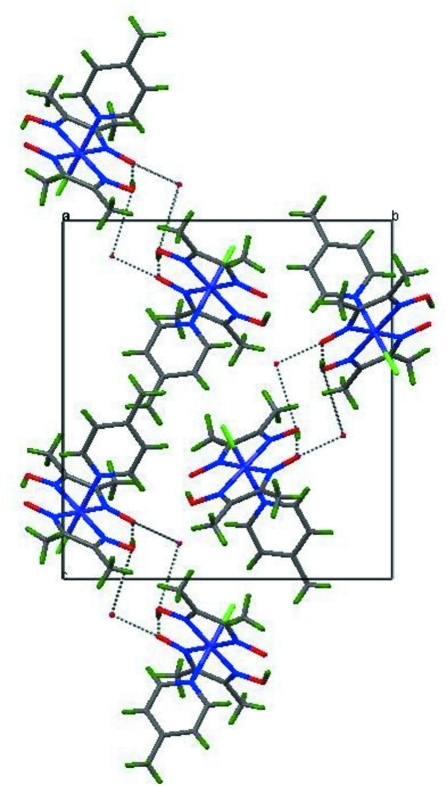
Packing of complex in the unit cell.

## supplementary crystallographic information

### Comment

Cobaloximes have been used extensively as structural and functional mimics for vitamin-B_12_ (Brown, 2005 and Randaccio *et al.*, 1989).Their chemical properties have been widely studied (Schrauzer *et al.*,1966).The two aspects of cobaloxime chemistry are their (*a*) inertness with respect to ligand exchange making them to serve as ideal system for studies relating to electron transfer reactions and (*b*)crystal parameters.The distance between glyoximato oxygen atoms in these complexes amount to 2.4–2.6 Å, a distance range of considerable interest for strong intra molecular hydrogen bonding (Reemers *et al.*, 2002).Compared to cobalamins, cobaloximes have shorter Co—N axial bond distance. It is known that coenzymes are related to number of 1,2-intra molecular rearrangement reactions (Dolphin *et al.*,1982).Most of the recent studies on cobaloximes have been focused on their structure–property relationships (Gupta *et al.*, 2004 and Dutta *et al.*, 2009).

In this title complex, the coordination about the Co^III^ ion is slightly distorted octahedral (Revathi *et al.*, 2009 and Kavitha *et al.*, 2008) with the 4-methylpyridine and chloride ligands occupying the axial positions and the two dimethylglyoximato ligands occupying the equatorial sites. The bite angles N1—Co—N2 and N3—Co—N4 of the ligand are 81.45 (8)^0^ and 81.17 (8)°, respectively.The coordinated chloride and the pyridine ring nitrogen are collinear with cobalt(III) froming an axial bond angle [N5—Co—Cl] = 178.89 (5)° and are perpendicular to the equatorial plane.The two glyoximate moiteies are linked together by strong inter molecular hydrogen bonding.

### Experimental

The complex was prepared by the literature method (Schrauzer *et al.*,1966) using H[Co(dmgH)_2_ Cl_2_] as the starting material (Ramesh *et al.*,2008). The dichloro cobaloxime was mixed with 4-methylpyridine in 1:1 molar ratio in about 60 ml of ethanol and allowed to stir for 3 hrs. The resulting brown coloured complex was filtered,washed with absolute ethanol followed by ether and dried over vacuum desicator. Crystals of the complex were grown in ethanol by slow evaporation method. The complex was characterized by UV, IR and H^1^NMR spectra. A moderately intense band around 250 nm may be ascribed to π- π* transition of the dmgH ^-^ group. A shoulder around 330 nm may be due to the ligand to metal charge transfer transition, LMCT (Dayalan *et al.*, 2001). The C=N stretching vibration of the oxime in its complex was observed at 1580 cm^-1^ and the intra molecular hydrogen bonded –OH around 3450 cm^-1^. A moderate peak at 1094 cm^-1^ may be assigned to the C=N—O stretching of the oxime. The peak at 513 cm^-1^ could be attributed to cobalt(III)-nitrogen stretching (Bline *et al.*,1958). The ^1^H NMR spectrum of the complex in DMSO-d_6_ shows a sharp intense singlet at 2.4 p.p.m. corresponding to methyl protons of the dimethylglyoximate. A singlet at 3.2 p.p.m. may be due to methyl protons of axial 4-methylpyridine ligand. The oxime –OH proton resonates at 8.28 p.p.m.. The two doublets at 8.07 and 7.4 p.p.m. correspond to pyridine ring protons at 2 & 6 and 3 & 5 positions, respectively of 4-methylpyridine at the axial position of the complex (Silverstein *et al.*,1984).

### Refinement

All the hydrogen atoms were identified from the difference electron density peak and fixed accordingly. The H atom bound to methyl C atoms were constrained to riding atoms wit d(C—H) = 0.96Å and *U*_iso_(H) = 1.5*U*_equ_(C). and the hydrogen atoms bound to aromatic carbon were constrained to riding atoms with d(C—H) = 0.93Å and *U*_iso_(H) =1.2*U*_equ_(C).The posotion of the hydrogen atom bound to the hydroxyl group was identified from the difference in the electron density map and restrained to a distance of d(O2—H2) = 0.90 (1) Å. The lattice solvent water O5 is left as anisotropically refined without the hydrogen being fixed but the water hydrogen is included in the chemical formula.

### Crystal data

**Table:**

[Co(C_4_H_7_N_2_O_2_)_2_Cl(C_6_H_7_N)]·0.5H_2_O	*D*_x_ = 1.512 Mg m^−^^3^
*M**_r_* = 425.74	Mo *K*α radiation, λ = 0.71073 Å
Orthorhombic, *P*2_1_2_1_2_1_	Cell parameters from 5528 reflections
*a* = 8.330 (5) Å	θ = 2.6–28.2°
*b* = 14.365 (5) Å	µ = 1.09 mm^−^^1^
*c* = 15.634 (5) Å	*T* = 293 K
*V* = 1870.8 (14) Å^3^	Block, brown
*Z* = 4	0.25 × 0.20 × 0.20 mm
*F*(000) = 880	

### Data collection

**Table:**

Bruker SMART APEXII CCD diffractometer	4642 independent reflections
Radiation source: fine-focus sealed tube	4143 reflections with *I* > 2σ(*I*)
graphite	*R*_int_ = 0.028
ω and φ scans	θ_max_ = 28.4°, θ_min_ = 1.9°
Absorption correction: multi-scan (*SADABS*; Bruker 2008)	*h* = −10→10
*T*_min_ = 0.772, *T*_max_ = 0.811	*k* = −19→18
10519 measured reflections	*l* = −20→20

### Refinement

**Table:**

Refinement on *F*^2^	Secondary atom site location: difference Fourier map
Least-squares matrix: full	Hydrogen site location: inferred from neighbouring sites
*R*[*F*^2^ > 2σ(*F*^2^)] = 0.029	H atoms treated by a mixture of independent and constrained refinement
*wR*(*F*^2^) = 0.077	*w* = 1/[σ^2^(*F*_o_^2^) + (0.0405*P*)^2^] where *P* = (*F*_o_^2^ + 2*F*_c_^2^)/3
*S* = 1.02	(Δ/σ)_max_ = 0.001
4642 reflections	Δρ_max_ = 0.40 e Å^−^^3^
248 parameters	Δρ_min_ = −0.38 e Å^−^^3^
2 restraints	Absolute structure: Flack (1983), 1983 Friedel pairs
Primary atom site location: structure-invariant direct methods	Flack parameter: 0.014 (13)

### Special details

**Table:**

Geometry. All e.s.d.'s (except the e.s.d. in the dihedral angle between two l.s. planes) are estimated using the full covariance matrix. The cell e.s.d.'s are taken into account individually in the estimation of e.s.d.'s in distances, angles and torsion angles; correlations between e.s.d.'s in cell parameters are only used when they are defined by crystal symmetry. An approximate (isotropic) treatment of cell e.s.d.'s is used for estimating e.s.d.'s involving l.s. planes.
Refinement. Refinement of *F*^2^ against ALL reflections. The weighted *R*-factor *wR* and goodness of fit *S* are based on *F*^2^, conventional *R*-factors *R* are based on *F*, with *F* set to zero for negative *F*^2^. The threshold expression of *F*^2^ > σ(*F*^2^) is used only for calculating *R*-factors(gt) *etc*. and is not relevant to the choice of reflections for refinement. *R*-factors based on *F*^2^ are statistically about twice as large as those based on *F*, and *R*- factors based on ALL data will be even larger.

### Fractional atomic coordinates and isotropic or equivalent isotropic displacement parameters (Å^2^)

**Table:**

	*x*	*y*	*z*	*U*_iso_*/*U*_eq_	Occ. (<1)
C1	0.7398 (3)	0.39874 (18)	0.25266 (16)	0.0398 (5)	
C2	0.7058 (3)	0.49050 (18)	0.28872 (15)	0.0408 (6)	
C3	0.8891 (3)	0.3435 (2)	0.2701 (2)	0.0643 (9)	
H3A	0.8637	0.2783	0.2702	0.097*	
H3B	0.9319	0.3608	0.3249	0.097*	
H3C	0.9672	0.3560	0.2265	0.097*	
C4	0.8147 (4)	0.5421 (3)	0.3478 (2)	0.0741 (10)	
H4A	0.7709	0.6026	0.3591	0.111*	
H4B	0.9185	0.5485	0.3218	0.111*	
H4C	0.8248	0.5083	0.4005	0.111*	
C5	0.1881 (3)	0.40417 (17)	0.08949 (15)	0.0406 (6)	
C6	0.1538 (3)	0.49521 (17)	0.12558 (16)	0.0392 (5)	
C7	0.0753 (4)	0.3542 (3)	0.0314 (2)	0.0759 (10)	
H7A	0.0627	0.3891	−0.0206	0.114*	
H7B	−0.0270	0.3477	0.0589	0.114*	
H7C	0.1177	0.2937	0.0183	0.114*	
C8	0.0047 (3)	0.5485 (3)	0.1072 (2)	0.0644 (9)	
H8A	0.0054	0.6055	0.1393	0.097*	
H8B	−0.0870	0.5121	0.1232	0.097*	
H8C	−0.0004	0.5625	0.0472	0.097*	
C9	0.2872 (3)	0.43850 (15)	0.35158 (13)	0.0331 (4)	
H9	0.2853	0.5030	0.3467	0.040*	
C10	0.2280 (3)	0.39842 (16)	0.42473 (15)	0.0387 (5)	
H10	0.1854	0.4358	0.4677	0.046*	
C11	0.2313 (3)	0.30297 (17)	0.43490 (15)	0.0376 (5)	
C12	0.2910 (3)	0.25176 (16)	0.36721 (16)	0.0376 (5)	
H12	0.2935	0.1871	0.3707	0.045*	
C13	0.3464 (3)	0.29497 (15)	0.29503 (15)	0.0351 (5)	
H13	0.3846	0.2588	0.2501	0.042*	
C14	0.1738 (4)	0.2569 (2)	0.51559 (18)	0.0596 (8)	
H14A	0.0587	0.2530	0.5148	0.089*	
H14B	0.2075	0.2928	0.5641	0.089*	
H14C	0.2184	0.1954	0.5193	0.089*	
N1	0.6300 (2)	0.36941 (12)	0.20091 (12)	0.0341 (4)	
N2	0.5693 (3)	0.52355 (12)	0.26275 (11)	0.0349 (4)	
N3	0.3268 (3)	0.37229 (13)	0.11255 (12)	0.0346 (4)	
N4	0.2679 (2)	0.52672 (12)	0.17406 (12)	0.0334 (4)	
N5	0.3474 (2)	0.38848 (11)	0.28726 (11)	0.0269 (4)	
O1	0.6426 (2)	0.28837 (12)	0.15990 (12)	0.0484 (4)	
O2	0.5187 (3)	0.60675 (11)	0.28888 (12)	0.0476 (5)	
O3	0.3788 (3)	0.29032 (14)	0.08378 (12)	0.0524 (5)	
O4	0.2592 (2)	0.61073 (11)	0.21081 (12)	0.0483 (5)	
Cl1	0.56856 (8)	0.51419 (4)	0.07531 (4)	0.04044 (14)	
Co1	0.44843 (3)	0.447668 (17)	0.188051 (17)	0.02570 (8)	
O5	0.8676 (9)	0.1476 (5)	0.1002 (5)	0.117 (2)	0.50
H3	0.4797 (19)	0.288 (2)	0.104 (2)	0.066 (11)*	
H2	0.426 (3)	0.621 (3)	0.261 (2)	0.087 (13)*	

### Atomic displacement parameters (Å^2^)

**Table:**

	*U*^11^	*U*^22^	*U*^33^	*U*^12^	*U*^13^	*U*^23^
C1	0.0293 (12)	0.0521 (14)	0.0381 (12)	−0.0008 (10)	−0.0037 (10)	0.0140 (11)
C2	0.0345 (12)	0.0535 (14)	0.0345 (12)	−0.0124 (11)	−0.0104 (10)	0.0099 (10)
C3	0.0362 (14)	0.080 (2)	0.076 (2)	0.0095 (14)	−0.0095 (14)	0.0267 (18)
C4	0.069 (2)	0.090 (2)	0.0638 (19)	−0.023 (2)	−0.0352 (17)	0.0010 (18)
C5	0.0370 (13)	0.0506 (13)	0.0341 (12)	−0.0111 (11)	−0.0101 (10)	0.0045 (10)
C6	0.0277 (12)	0.0510 (13)	0.0388 (12)	0.0003 (11)	−0.0027 (10)	0.0144 (10)
C7	0.071 (2)	0.089 (2)	0.068 (2)	−0.024 (2)	−0.0365 (18)	−0.0029 (18)
C8	0.0343 (14)	0.087 (2)	0.0724 (19)	0.0160 (14)	−0.0032 (13)	0.0242 (18)
C9	0.0363 (11)	0.0300 (10)	0.0331 (10)	−0.0003 (9)	0.0018 (9)	−0.0065 (9)
C10	0.0412 (13)	0.0441 (12)	0.0309 (10)	−0.0013 (10)	0.0052 (11)	−0.0101 (10)
C11	0.0328 (12)	0.0482 (13)	0.0317 (11)	−0.0059 (10)	−0.0030 (10)	0.0040 (10)
C12	0.0405 (13)	0.0306 (10)	0.0415 (12)	0.0002 (10)	0.0020 (11)	0.0042 (9)
C13	0.0402 (12)	0.0278 (9)	0.0372 (12)	0.0029 (9)	0.0023 (10)	−0.0032 (8)
C14	0.067 (2)	0.0712 (18)	0.0405 (16)	−0.0139 (16)	0.0089 (15)	0.0088 (14)
N1	0.0303 (9)	0.0373 (9)	0.0348 (10)	0.0051 (7)	0.0020 (8)	0.0029 (8)
N2	0.0413 (11)	0.0320 (8)	0.0312 (9)	−0.0089 (9)	−0.0018 (9)	−0.0001 (7)
N3	0.0401 (12)	0.0355 (9)	0.0283 (9)	−0.0036 (8)	−0.0025 (8)	−0.0054 (7)
N4	0.0344 (10)	0.0315 (8)	0.0343 (10)	0.0049 (7)	0.0001 (8)	0.0037 (7)
N5	0.0273 (9)	0.0251 (7)	0.0282 (8)	0.0018 (7)	−0.0005 (7)	−0.0013 (6)
O1	0.0480 (10)	0.0418 (8)	0.0553 (11)	0.0150 (8)	0.0079 (9)	−0.0052 (8)
O2	0.0639 (13)	0.0307 (7)	0.0482 (10)	−0.0095 (8)	−0.0048 (9)	−0.0084 (7)
O3	0.0678 (13)	0.0433 (9)	0.0463 (11)	0.0021 (9)	−0.0060 (10)	−0.0203 (8)
O4	0.0586 (12)	0.0314 (8)	0.0549 (11)	0.0134 (8)	0.0025 (9)	−0.0017 (8)
Cl1	0.0367 (3)	0.0529 (3)	0.0317 (2)	−0.0044 (3)	−0.0007 (3)	0.0089 (2)
Co1	0.02577 (13)	0.02728 (12)	0.02404 (12)	0.00065 (11)	−0.00294 (11)	−0.00153 (10)
O5	0.119 (5)	0.121 (4)	0.111 (5)	0.036 (5)	0.008 (4)	0.015 (4)

### Geometric parameters (Å, °)

**Table:**

C1—N1	1.292 (3)	C9—H9	0.9300
C1—C2	1.461 (4)	C10—C11	1.381 (3)
C1—C3	1.500 (3)	C10—H10	0.9300
C2—N2	1.297 (3)	C11—C12	1.382 (3)
C2—C4	1.491 (4)	C11—C14	1.503 (3)
C3—H3A	0.9600	C12—C13	1.368 (3)
C3—H3B	0.9600	C12—H12	0.9300
C3—H3C	0.9600	C13—N5	1.349 (3)
C4—H4A	0.9600	C13—H13	0.9300
C4—H4B	0.9600	C14—H14A	0.9600
C4—H4C	0.9600	C14—H14B	0.9600
C5—N3	1.294 (3)	C14—H14C	0.9600
C5—C6	1.453 (4)	N1—O1	1.333 (3)
C5—C7	1.491 (4)	N1—Co1	1.8951 (19)
C6—N4	1.297 (3)	N2—O2	1.332 (3)
C6—C8	1.487 (4)	N2—Co1	1.8885 (19)
C7—H7A	0.9600	N3—O3	1.333 (3)
C7—H7B	0.9600	N3—Co1	1.8953 (19)
C7—H7C	0.9600	N4—O4	1.339 (2)
C8—H8A	0.9600	N4—Co1	1.897 (2)
C8—H8B	0.9600	N5—Co1	1.9589 (18)
C8—H8C	0.9600	O2—H2	0.907 (10)
C9—N5	1.334 (3)	O3—H3	0.900 (10)
C9—C10	1.372 (3)	Cl1—Co1	2.2408 (8)
			
O1···O5	2.911 (8)	O5···O3^i^	3.013 (7)
			
N1—C1—C2	113.5 (2)	C13—C12—C11	120.8 (2)
N1—C1—C3	121.9 (3)	C13—C12—H12	119.6
C2—C1—C3	124.6 (2)	C11—C12—H12	119.6
N2—C2—C1	112.3 (2)	N5—C13—C12	121.9 (2)
N2—C2—C4	123.0 (3)	N5—C13—H13	119.1
C1—C2—C4	124.7 (3)	C12—C13—H13	119.1
C1—C3—H3A	109.5	C11—C14—H14A	109.5
C1—C3—H3B	109.5	C11—C14—H14B	109.5
H3A—C3—H3B	109.5	H14A—C14—H14B	109.5
C1—C3—H3C	109.5	C11—C14—H14C	109.5
H3A—C3—H3C	109.5	H14A—C14—H14C	109.5
H3B—C3—H3C	109.5	H14B—C14—H14C	109.5
C2—C4—H4A	109.5	C1—N1—O1	122.0 (2)
C2—C4—H4B	109.5	C1—N1—Co1	115.98 (16)
H4A—C4—H4B	109.5	O1—N1—Co1	122.00 (15)
C2—C4—H4C	109.5	C2—N2—O2	120.7 (2)
H4A—C4—H4C	109.5	C2—N2—Co1	116.73 (17)
H4B—C4—H4C	109.5	O2—N2—Co1	122.61 (17)
N3—C5—C6	112.7 (2)	C5—N3—O3	120.6 (2)
N3—C5—C7	124.2 (3)	C5—N3—Co1	116.65 (17)
C6—C5—C7	123.1 (3)	O3—N3—Co1	122.76 (18)
N4—C6—C5	113.4 (2)	C6—N4—O4	121.7 (2)
N4—C6—C8	123.0 (3)	C6—N4—Co1	116.06 (16)
C5—C6—C8	123.6 (2)	O4—N4—Co1	122.21 (15)
C5—C7—H7A	109.5	C9—N5—C13	117.81 (19)
C5—C7—H7B	109.5	C9—N5—Co1	121.65 (14)
H7A—C7—H7B	109.5	C13—N5—Co1	120.40 (15)
C5—C7—H7C	109.5	N2—O2—H2	109 (2)
H7A—C7—H7C	109.5	N3—O3—H3	103 (2)
H7B—C7—H7C	109.5	N2—Co1—N1	81.44 (9)
C6—C8—H8A	109.5	N2—Co1—N3	179.57 (9)
C6—C8—H8B	109.5	N1—Co1—N3	98.84 (9)
H8A—C8—H8B	109.5	N2—Co1—N4	98.54 (9)
C6—C8—H8C	109.5	N1—Co1—N4	179.31 (9)
H8A—C8—H8C	109.5	N3—Co1—N4	81.17 (9)
H8B—C8—H8C	109.5	N2—Co1—N5	89.43 (8)
N5—C9—C10	122.5 (2)	N1—Co1—N5	90.09 (8)
N5—C9—H9	118.7	N3—Co1—N5	90.89 (8)
C10—C9—H9	118.7	N4—Co1—N5	90.60 (8)
C9—C10—C11	120.4 (2)	N2—Co1—Cl1	90.12 (6)
C9—C10—H10	119.8	N1—Co1—Cl1	88.85 (7)
C11—C10—H10	119.8	N3—Co1—Cl1	89.57 (7)
C10—C11—C12	116.6 (2)	N4—Co1—Cl1	90.46 (6)
C10—C11—C14	121.9 (2)	N5—Co1—Cl1	178.90 (6)
C12—C11—C14	121.5 (2)		
			
N1—C1—C2—N2	1.4 (3)	C2—N2—Co1—Cl1	−90.64 (17)
C3—C1—C2—N2	179.3 (2)	O2—N2—Co1—Cl1	89.93 (16)
N1—C1—C2—C4	−177.8 (3)	C1—N1—Co1—N2	2.65 (17)
C3—C1—C2—C4	0.1 (4)	O1—N1—Co1—N2	−178.23 (18)
N3—C5—C6—N4	−0.3 (3)	C1—N1—Co1—N3	−177.67 (17)
C7—C5—C6—N4	−179.6 (2)	O1—N1—Co1—N3	1.44 (18)
N3—C5—C6—C8	178.2 (2)	C1—N1—Co1—N4	91 (8)
C7—C5—C6—C8	−1.1 (4)	O1—N1—Co1—N4	−89 (8)
N5—C9—C10—C11	1.1 (4)	C1—N1—Co1—N5	−86.76 (17)
C9—C10—C11—C12	−2.2 (4)	O1—N1—Co1—N5	92.35 (17)
C9—C10—C11—C14	177.4 (2)	C1—N1—Co1—Cl1	92.94 (17)
C10—C11—C12—C13	1.3 (4)	O1—N1—Co1—Cl1	−87.94 (17)
C14—C11—C12—C13	−178.4 (2)	C5—N3—Co1—N2	−50 (13)
C11—C12—C13—N5	0.9 (4)	O3—N3—Co1—N2	130 (13)
C2—C1—N1—O1	177.96 (19)	C5—N3—Co1—N1	179.18 (17)
C3—C1—N1—O1	0.0 (3)	O3—N3—Co1—N1	−0.80 (19)
C2—C1—N1—Co1	−2.9 (3)	C5—N3—Co1—N4	−1.52 (17)
C3—C1—N1—Co1	179.11 (19)	O3—N3—Co1—N4	178.50 (19)
C1—C2—N2—O2	−179.79 (19)	C5—N3—Co1—N5	88.95 (18)
C4—C2—N2—O2	−0.5 (4)	O3—N3—Co1—N5	−91.03 (18)
C1—C2—N2—Co1	0.8 (3)	C5—N3—Co1—Cl1	−92.05 (17)
C4—C2—N2—Co1	−180.0 (2)	O3—N3—Co1—Cl1	87.97 (18)
C6—C5—N3—O3	−178.62 (19)	C6—N4—Co1—N2	−178.99 (16)
C7—C5—N3—O3	0.7 (4)	O4—N4—Co1—N2	2.02 (18)
C6—C5—N3—Co1	1.4 (3)	C6—N4—Co1—N1	92 (8)
C7—C5—N3—Co1	−179.3 (2)	O4—N4—Co1—N1	−87 (8)
C5—C6—N4—O4	178.05 (19)	C6—N4—Co1—N3	1.33 (16)
C8—C6—N4—O4	−0.4 (3)	O4—N4—Co1—N3	−177.66 (18)
C5—C6—N4—Co1	−0.9 (3)	C6—N4—Co1—N5	−89.47 (17)
C8—C6—N4—Co1	−179.41 (19)	O4—N4—Co1—N5	91.53 (17)
C10—C9—N5—C13	1.1 (3)	C6—N4—Co1—Cl1	90.82 (16)
C10—C9—N5—Co1	−174.48 (18)	O4—N4—Co1—Cl1	−88.17 (16)
C12—C13—N5—C9	−2.1 (4)	C9—N5—Co1—N2	47.81 (18)
C12—C13—N5—Co1	173.54 (18)	C13—N5—Co1—N2	−127.67 (19)
C2—N2—Co1—N1	−1.82 (17)	C9—N5—Co1—N1	129.25 (18)
O2—N2—Co1—N1	178.75 (18)	C13—N5—Co1—N1	−46.23 (18)
C2—N2—Co1—N3	−133 (13)	C9—N5—Co1—N3	−131.90 (18)
O2—N2—Co1—N3	47 (13)	C13—N5—Co1—N3	52.61 (18)
C2—N2—Co1—N4	178.87 (17)	C9—N5—Co1—N4	−50.73 (18)
O2—N2—Co1—N4	−0.56 (18)	C13—N5—Co1—N4	133.79 (18)
C2—N2—Co1—N5	88.36 (17)	C9—N5—Co1—Cl1	114 (3)
O2—N2—Co1—N5	−91.07 (17)	C13—N5—Co1—Cl1	−62 (3)

Symmetry codes: (i) *x*+1/2, −*y*+1/2, −*z*.

### Hydrogen-bond geometry (Å, °)

**Table:**

*D*—H···*A*	*D*—H	H···*A*	*D*···*A*	*D*—H···*A*
O3—H3···O1	0.90 (1)	1.61 (1)	2.499 (3)	168 (3)
O2—H2···O4	0.91 (1)	1.60 (2)	2.483 (3)	162 (4)
